# Beyond the game: evaluating a soccer-centered approach to physical activity and health education in children

**DOI:** 10.3389/fspor.2026.1765167

**Published:** 2026-07-15

**Authors:** Jordan D. Foster, Eva Li, Arturo Juarez, Jordan Pelkmans, Staci Fluellen, Kevin Li, Jocelyn Lee, Leilany Munoz, Siddhanth Rohith, Peter Krustrup, Malte N. Larsen, Jennifer K. Frediani

**Affiliations:** 1 Rollins School of Public Health, Emory University, Atlanta, GA, United States; 2 Emory College of Arts and Humanities, Emory University, Atlanta, GA, United States; 3 Nell Woodruff Hodgson School of Nursing, Emory University, Atlanta, GA, United States; 4 School of Public Health, Georgia State University, Atlanta, GA, United States; 5 Alliance Academy for Innovation, Cumming, GA, United States; 6 Department of Sports Science and Clinical Biomechanics, University of Southern Denmark, Odense, Denmark; 7 Sport and Health Sciences, University of Exeter, Exeter, United Kingdom

**Keywords:** soccer, association football, children, physical fitness, health knowledge, health literacy

## Abstract

**Background/objectives:**

Due to only 25% of U.S. children meeting the physical activity guidelines, we aimed to determine feasibility of team sports to engage children in at least 60 min of moderate-to-vigorous physical activity daily and increase health knowledge.

**Methods:**

This 11-week quasi-experimental pre/post intervention study was conducted in two after-school programs around metro Atlanta, GA, USA (C1, *n* = 18; C2, *n* = 22). The 11 for Health program is an 11-week active learning model that involves 2 × 45 min per week soccer drills, small-sided games, and health education modules. Assessments included health knowledge and physical fitness metrics such as estimated VO_2_, standing long jump, balance, agility, and handgrip strength. Non-participants were defined as missing 3 or more sessions and/or not participating in the activities. Descriptive statistics and t-tests were used to compare pre- and post-intervention assessments, stratified by participation level.

**Results:**

C1 were on average 9.2 years old, 31% female, and 100% White. C2 were on average 9.8 years, 48% female, and 67% Hispanic and 33% Black. Attendance was 69% (SD 0.26) in average over the 11 weeks for both cohorts, with no difference in attendance between groups. Agility improved significantly [2.7 s(1.5); 95% CI: −3.2,−2.2] overall but no between-group difference was found. C2 participants improved significantly compared to C1 participants (*p* = 0.006) in left handgrip. C1 performed significantly better on the health knowledge test (*p* = 0.023; 95% CI: 0.018, 0.225) but neither cohort improved over time.

**Conclusion:**

The program is feasible for American children; however, careful consideration of setting and coaching staff is necessary.

## 1 Introduction

Research on health literacy and physical activity interventions aiming at elementary school children in the United States is still evolving, partly due to constraints imposed by rigid curriculum standards (1). Despite these challenges, the integration of physical activity with health literacy interventions presents a promising strategy for enhancing children's health outcomes (1).

Physical activity interventions are linked to a range of benefits for children, including improved physical health, cognitive function, and psychological well-being. The Centers for Disease Control and Prevention (CDC) recommends that children should engage in at least 60 min of moderate-to-vigorous physical activity daily (2). Regular physical activity is associated with enhanced academic performance, improved attention, better mood regulation, and a reduction in symptoms of anxiety and depression (3–5). However, the CDC reported that less than one-quarter (24%) of children meet this requirement (2, 6).

Numerous strategies exist to increase children's daily physical activity, with organized recreational team sports serving as a viable option. Health literacy is also vital for empowering children to make informed health choices. Effective health literacy programs tailored for children can significantly enhance their understanding of health concepts, leading to improved health behaviors. When combined with physical activity interventions, these programs create a more comprehensive approach to health promotion (7–9).

The “11 for Health” program is an initiative established by the Fédération Internationale de Football Association (FIFA) Medical Assessment and Research Centre (F-MARC) in 2009, designed to promote physical activity among children through soccer-based sessions while integrating education on prevalent health issues and launched in 2010 in sub-Saharan Africa, the program combined soccer skills with health education, focusing primarily on infectious diseases (10). Danish researchers adapted the program in 2015 for schools in Europe, incorporating twice-weekly 45 min sessions with periods of vigorous exercise, and additional topics related to nutrition and exercise to address common health problems (11). Evaluations of the adaptation have shown positive impacts on participants' physical health, overall well-being, and health knowledge in Danish and Faroese children, including ethnic minority, socially vulnerable and overweight children (12, 13). A key modification in the American version of the program includes the incorporation of weight-neutral strategies. For example, the session previously titled “Controlling Your Weight” was renamed “Managing Your Energy” to emphasize fueling the body for exercise rather than concentrating on weight maintenance or loss. This weight-neutral approach aims to mitigate weight stigma while encouraging a variety of foods and daily physical activity.

The U.S. faces multiple challenges when implementing school-based interventions during the school day. The typical school day lasts approximately seven hours, five days a week, yet students receive an average of only two physical education sessions per week, each lasting about 50 min. Time constraints driven by strict curriculum requirements often limit the feasibility of adding new health promotion activities during school hours. In addition, schools serving low-income, racially and ethnically diverse communities frequently face systemic barriers that reduce students' access to extracurricular opportunities such as after-school and physical activity programs. These barriers may include limited school funding, transportation difficulties, program costs, and reduced parental availability (14, 15). As a result, schools in underserved areas often have fewer opportunities and resources to provide physical education, extracurricular sports, or structured wellness initiatives compared to schools in more affluent communities. Given these challenges, this study chose to implement the intervention in two different after-school programs. After-school settings offer students safe, structured environments beyond the traditional school day and often reach children who might otherwise lack opportunities for consistent physical activity. The first location was an after-school setting provided by an upper elementary school in an affluent area of metro Atlanta. This particular after-school program operates on a choice-based model, allowing children to select from diverse activities such as homework, board games, arts and crafts, reading, outdoor play, food projects, drama, and quiet games (16). The second location was a community organization that provides an after-school program to 225K-12 students from nine partnering schools in an underserved part of metro Atlanta. This after-school program provides transportation, tutoring and homework assistance, mentorship, educational and recreational activities, standardized test preparation, physical fitness, and meals for the kids (17). Both settings offered unique and comparable environments for applying the 11 for Health program and assessing its impact in a real-world context. Therefore, the objective of this observational study was to assess the feasibility of implementing the 11 for Health program among upper elementary school children participating in an after-school program in the United States. Feasibility was defined as successful delivery of all sessions and baseline and end of study assessments. We hypothesized that this integrated soccer-based program would be feasible to deliver and enhance health knowledge and improve physical fitness. This report presents our findings and insights regarding the implementation of this health behavior program for 8- to 11-year-olds within the two different after-school settings.

## 2 Materials and methods

This study was conducted in two different cohorts using an exploratory pre/post quasi experimental design. Both cohorts were conducted in an after-school care program around the Atlanta metro area in children aged 8–11 years old. All children and their parents enrolled in each program were invited to participate. There were no exclusion criteria.

Cohort 1 (C1) was conducted in a school located in a small city near Atlanta, GA, with a population of 24,338. The demographic composition of the area includes 66% non-Hispanic White, 15% non-Hispanic Black, and 4% Asian. The average household income in the region is $129,992, according to 2022 data (18). There was a total of 75 children enrolled. Of these, 21 expressed interests, and ultimately, 18 were enrolled in the study. Among the 18 enrolled participants, 13 completed both the baseline and end-of-study assessments. C1 was conducted from August 2023, which included recruitment and consent procedures, to December 2023, when end-of-study assessments were completed. Two week-long school breaks occurred in September and November. The first week of the intervention commenced after the September break, and the tenth week concluded in the second week of December.

Due to field reseeding and scheduled school breaks, C1 was shortened to 10 weeks, concluding before the holiday break. Table 1 outlines the soccer and public health topics covered each week. The originally planned Week 11, intended for summarizing health messages and conducting small-sided games, was omitted. Instead, the summary was integrated into the final session of Week 10, ensuring that all 10 planned health message sessions were successfully completed for C1.

**Table 1:** Weekly intervention topics.

Week	Soccer activity	Health message
1	Warming up	Play Soccer
2	Passing	Respect others
3	Goalkeeping	Be active
4	Dribbling	Avoid drugs, alcohol, tobacco
5	Controlling the ball	Managing your energy
6	Defending	Wash your hands
7	Trapping	Drink water
8	Building fitness	Eat a balanced diet
9	Overlapping	Get stronger
10	Shooting	Think positively
11	Wrap up	Summary

Cohort 2 (C2) was in an after-school program that services three different Atlanta elementary schools located on the west side. The after-school population all qualify for free or reduced lunch and are 90% Hispanic and 10% African American. There was a total of 59 children (8–11-year-olds) enrolled in the after-school program, 27 interested in the program and ultimately 22 enrolled, with 21 completing both baseline and end of study measurements. C2 was conducted in January 2025 with recruitment and ended in May 2025 with end of study measurements. This cohort completed all 11 weeks and had week-long breaks for winter and spring break.

### 2.1 Intervention

The “11 for Health” program follows the social constructivist learning model with experiential learning activities. It is an 11-week intervention designed to teach soccer skills alongside public health messages to elementary school-aged children. Key modifications from previous international implementations involved changing session topics from “Controlling Your Weight” and “Being Fit” to “Managing Your Energy” and “Getting Stronger”, respectively, to promote weight-neutral language and reduce the emphasis on weight loss among children.

Colleagues from Denmark conducted a two-day in-person training for the Principal Investigator (PI) and one student prior to the first cohort. The PI then trained all other student coaches. A comprehensive manual, which contained detailed educational sessions, warm-ups, and drills for each week, guided the intervention. The intervention was implemented by the PI and J.F., A.J. and S.F. for C1 and by students only unless unavailable for C2, E.L, K.L. and J.L. Fidelity was measured in C2. Sessions were selected at random to record using a video camcorder with a microphone on the primary student conducting the session that day. These videos were downloaded and reviewed by two different students that were not involved in the implementation of the program using a standardized checklist (L.M. and S.R.). The standardized checklist was developed for each type of session, soccer or health message, based on 12–15 behavior change techniques derived from the 11 for Health manual. Sessions were conducted on Mondays and Wednesdays, each lasting 45 min, and took place on an outdoor field at the school for C1. C2 was conducted on Wednesdays for 90 min in a large gym. These differences were due to scheduling of other programs concurrently conducted at the facilities. C1's facility did not provide any additional counselors to help with behavior issues; C2 provided a counselor for every session to help with the children.

### 2.2 Measurements

Baseline assessments were conducted as a group one week prior to the intervention and again the week following the final week of instruction in both cohorts. The test protocol included assessments of agility, handgrip strength, postural balance, horizontal jump performance, cardiorespiratory fitness, and evaluations of knowledge and program satisfaction.

#### 2.2.1 Physical fitness measures

##### 2.2.1.1 Agility

Agility was measured using the Arrowhead Agility test, which evaluates body control and directional changes, commonly used for soccer players (19). Four cones were set up, and students viewed a demonstration and completed a practice trial before being timed. Each student ran the course twice, with the lower of the two times recorded for analysis.

##### 2.2.1.2 Handgrip strength

Handgrip strength was assessed using a portable digital dynamometer (Takei Model T.K.K.540). After a demonstration, students performed three trials with each hand, alternating between hands. The average grip strength for each hand was calculated, and the dominant hand was recorded.

##### 2.2.1.3 Postural balance

The Standing Stork Balance Test was used to assess postural balance (20). Participants stood on one leg with the other foot resting on the knee of the standing leg. The test leader started a stopwatch when the participants lifted their heels off the ground to balance on the ball of their foot. Timing stopped if the heel touched the floor. Each leg was tested twice, with the maximum time recorded up to 60 s. Participants were allowed one familiarization attempt per leg before the official test.

##### 2.2.1.4 Standing long jump

The standing long jump was conducted on a sidewalk or in a gym where a tape measure was laid out with a clear starting line (21). Participants started from behind the line, adopted a squatting position, and jumped as far as possible, landing on both feet. The distance from the start line to the point where the rear heel landed was measured. Participants performed two jumps, with the longest distance recorded in centimeters. A familiarization jump was allowed before the official test.

##### 2.2.1.5 20 m multistage fitness test (beep test)

This test was conducted indoors in a hallway with markers placed 20 meters apart (22). Participants ran 20-meter intervals at speeds indicated by a beeping signal, with a 10 s recovery period between intervals. The speed increased as the test progressed until participants could no longer keep pace with the beeps. The level achieved was recorded, and the estimated VO_2_ max was calculated based on this level (23).

##### 2.2.1.6 Health knowledge questionnaire

Participants completed a 34-item health knowledge questionnaire on REDCap, under the supervision of study staff. The questionnaire assessed health knowledge through true/false and multiple-choice questions. Questions covering 10 health topics were randomly distributed. Three control questions were also included. These consisted of questions any child aged 8–11 years should be able to answer. Correct response rates and standard deviations for each health message were calculated from individual answers.

### 2.3 Statistical analysis

We analyzed physical fitness measurements by comparing values across different participation groups over time. Participants were categorized into “High Participation” and “Low Participation” groups based on their attendance consistency throughout the study. Participants with 3 or more absences and/or lack of participation in the activities were classified as “Low Participation”. Detailed notes were taken at the end of each session that noted who did not participate. For example, those participants that chose not to play the small sided soccer games by either sitting on the sideline or choosing to just stand off to the side for the majority of the session. For handgrip strength, two measurements were taken, and the average values were used in the analysis. In contrast, the lowest time recorded from two trials of the Arrowhead agility test was selected, and the maximum long jump and postural balance duration of two trials was used. Subsequently, we compared the results between high participation and low participation, separately for baseline and post-intervention assessments. Statistical comparisons were performed using Wilcoxon rank sum tests, including paired and independent, while descriptive statistics, including means, were employed to evaluate differences within each group (high vs. low participation) between baseline and post-intervention measurements. This analysis aimed to elucidate changes in physical fitness measurements over the course of the intervention for both groups. Wilcoxon effect sizes are also reported. All statistical analyses were conducted using R Version 2023.12.1 + 402.

## 3 Results

The average age of participants was nearly identical across cohorts [9.6 years (SD = 0.97)]. Cohort 2 (C2) had a higher proportion of female participants (48%) compared to Cohort 1 (C1) at 31%. All C1 participants identified as non-Hispanic White, whereas none in C2 did; instead, 67% identified as Hispanic and 33% as Black. Most children had prior soccer experience, 81% in C2% and 77% in C1, with no significant difference between groups (*p* > 0.05). A greater proportion of C1 participants were classified as having high participation (62%) compared to C2 (48%), though this difference was also not statistically significant (*p* > 0.05). All demographics can be found in Table 2.

**Table 2:** Demographics, overall and by cohort.

Characteristic	Overall *N* = 34^a^	95% CI	Cohort 1 *N* = 13^a^	95% CI	Cohort 2 *N* = 21^a^	95% CI	*p*-value^b^
Age (years)	9.58 (0.97)	9.2, 9.9	9.17 (0.68)	8.7, 9.6	9.83 (1.05)	9.3, 10	0.083
Unknown	2		1		1		
Female Sex	14 (41%)	25%, 59%	4 (31%)	10%, 61%	10 (48%)	26%, 70%	0.3
Race/Ethnicity							<0.001
White, Non-Hispanic	13 (38%)	23%, 56%	13 (100%)	72%, 100%	0 (0%)	0.00%, 19%	
White, Hispanic	13 (38%)	23%, 56%	0 (0%)	0.00%, 28%	13 (62%)	39%, 81%	
Black, Non-Hispanic	7 (21%)	9.3%, 38%	0 (0%)	0.00%, 28%	7 (33%)	15%, 57%	
Black, Hispanic	1 (2.9%)	0.15%, 17%	0 (0%)	0.00%, 28%	1 (4.8%)	0.25%, 26%	
Soccer Experience	27 (79%)	62%, 91%	10 (77%)	46%, 94%	17 (81%)	57%, 94%	>0.9
Participation							0.4
Non-Participant	18 (53%)	35%, 70%	8 (62%)	32%, 85%	10 (48%)	26%, 70%	
Participant	16 (47%)	30%, 65%	5 (38%)	15%, 68%	11 (52%)	30%, 74%	

CI , confidence interval.

a Mean (SD; *n* (%).

b Wilcoxon rank sum test; Pearson's Chi-squared test; Fisher's exact test.

**Figure 1:**
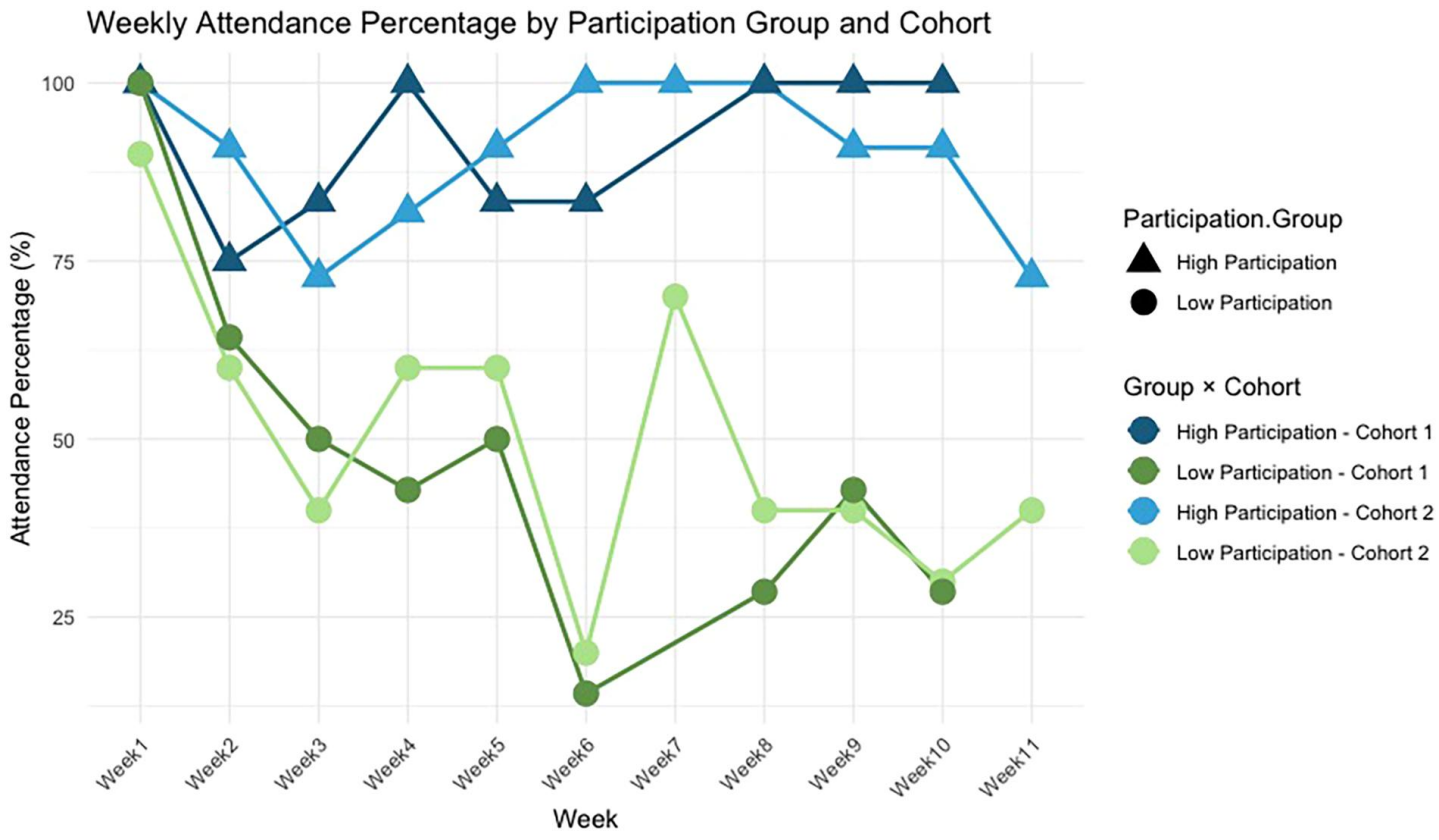
Attendance percentage over the 11-week intervention. The *x* axis states the week of the intervention, and the *y* axis states the average attendance percentage. The legend on the right distinguishes low and high participation and cohort: dark green with a circle line represents low participation in Cohort 1 and light green with a circle line represents low participation in Cohort 2, and dark blue with a triangle line represents high participation in Cohort 1 and light blue with a triangle line represents high participation in Cohort 2.

Figure 1 illustrates average attendance over time, stratified by cohort and participation level. High participants maintained consistent attendance throughout the 11 weeks, while non-participants showed a steady decline. Supplementary Figure S1 provides additional detail.

In C1, which held sessions twice weekly, attendance peaked in Week 1 at 100% on both days. It dipped slightly in Week 2 (∼95%) and declined further in Week 3 following the dropout of two children, with another leaving in Week 5. All dropouts agreed to complete end-of-study measurements. The lowest attendance occurred on Day 1 of Week 6 (∼30%), coinciding with a Halloween event. Attendance rebounded in Week 7 (50%–65%) and remained stable through Week 10. Overall, C1 showed an initial peak, mid-study decline, and partial recovery.

C2 never reached full attendance, though Week 1 had the highest rate with only one absence. Attendance dropped in Weeks 2 and 3, largely due to tutoring conflicts. Two children withdrew in Week 5, and two others had overlapping basketball practices. Attendance declined again in Weeks 10 and 11 (62% and 57%, respectively). C2 exhibited a similar pattern: early high attendance, mid-study decline, and a final drop. Overall, the data indicate an initial high attendance rate that decreased shortly after and remained relatively consistent before decreasing again at the end of the study.

### 3.1 Intention to treat analysis—physical activity measurements

Across all participants, significant improvements were observed from pre- to post-intervention in estimated VO₂ mL/min/kg [1.75 [2.81]; 95% CI [0.74, 2.80]; Wilcoxon Effect Size 0.79; *p*-value 0.001], agility [−2.7 [1.5]; CI [−3.2, −2.2]; Wilcoxon Effect Size 0.99; *p*-value < 0.001], and right leg balance [8 s (15); CI (2.3, 13); Wilcoxon Effect Size 0.57; *p*-value 0.011]. Cohort 2 was significantly higher than Cohort 1at baseline for estimated VO_2_ max and right handgrip strength. Cohort 1 had significantly higher balance times than Cohort 2 at baseline. These differences held at the end of study, in addition Cohort 1 had significantly higher standing long jump results (Table 3).

**Table 3:** Physical activity measurements for all participants at baseline and end of study.

	Baseline					End of Study				
Characteristics	Cohort 1^1^	Cohort 2^1^	Difference (95% CI)^2^	*p*-value^4^	Effect Size^5^	Cohort 1^1^	Cohort 2^1^	Difference (95% CI)^2^	*p*-value^4^	Effect Size^5^
Estimated VO_2_ (mL/min/kg)	17.59 (1.13)	24.12 (4.73)	6.53 (4.23, 8.82)	<0.001	0.87	18.83 (1.91)	26.17 (5.61)	7.34 (4.52, 10.16)	<0.001	0.90
Agility (sec)	10.90 (1.11)	11.66 (1.77)	0.76 (−0.28, 1.79)	0.195	0.28	8.36 (0.68)	8.85 (1.32)	0.49 (−0.24, 1.21)	0.272	0.24
Right Handgrip (kg)	12.55 (2.67)	15.51 (4.36)	2.96 (0.42, 5.49)	0.037	0.45	12.39 (2.17)	15.98 (4.56)	3.59 (1.14, 6.04)	0.009	0.56
Left Handgrip (kg)	12.71 (3.75)	13.77 (3.78)	1.06 (−1.77, 3.90)	0.521	0.14	12.90 (3.59)	14.56 (3.73)	1.66 (−1.09, 4.40)	0.251	0.25
Long Jump (cm)	164.26 (30.51)	148.94 (23.18)	−15.32 (−36.73, 6.10)	0.155	0.31	179.17 (37.63)	146.62 (24.12)	−32.55 (−58.20, −6.91)	0.009	0.56
Right Leg Balance (sec)	47.25 (16.88)	29.19 (17.99)	−18.06 (−31.09, −5.03)	0.006	0.58	52.64 (16.17)	38.15 (17.84)	−14.49 (−27.13, −1.85)	0.025	0.46
Left Leg Balance (sec)	55.83 (9.96)	31.47 (19.83)	−24.36 (−35.16, −13.56)	<0.001	0.71	55.44 (11.04)	37.79 (17.85)	−17.65 (−28.08, −7.22)	0.003	0.60

^1^Mean (SD); ^2^Difference in means (95% Confidence Interval); ^4^Wilcoxon rank sum test; Wilcoxon rank sum exact test; ^5^Wilcoxon Effect Size (Unpaired).

### 3.2 Cohort comparisons by participation level

Among high participants, C2 showed greater improvements in estimated VO₂, agility, and grip strength (both hands), though only left-hand grip strength reached statistical significance (C2- 0.62 kg (0.86); [0.00, 1.20]; C1- −1.16 kg (0.68); [−2.00, −0.32]; Wilcoxon Effect Size 0.92; *p* = 0.003). Leg balance improvements were similar across cohorts.

**Figure 2:**
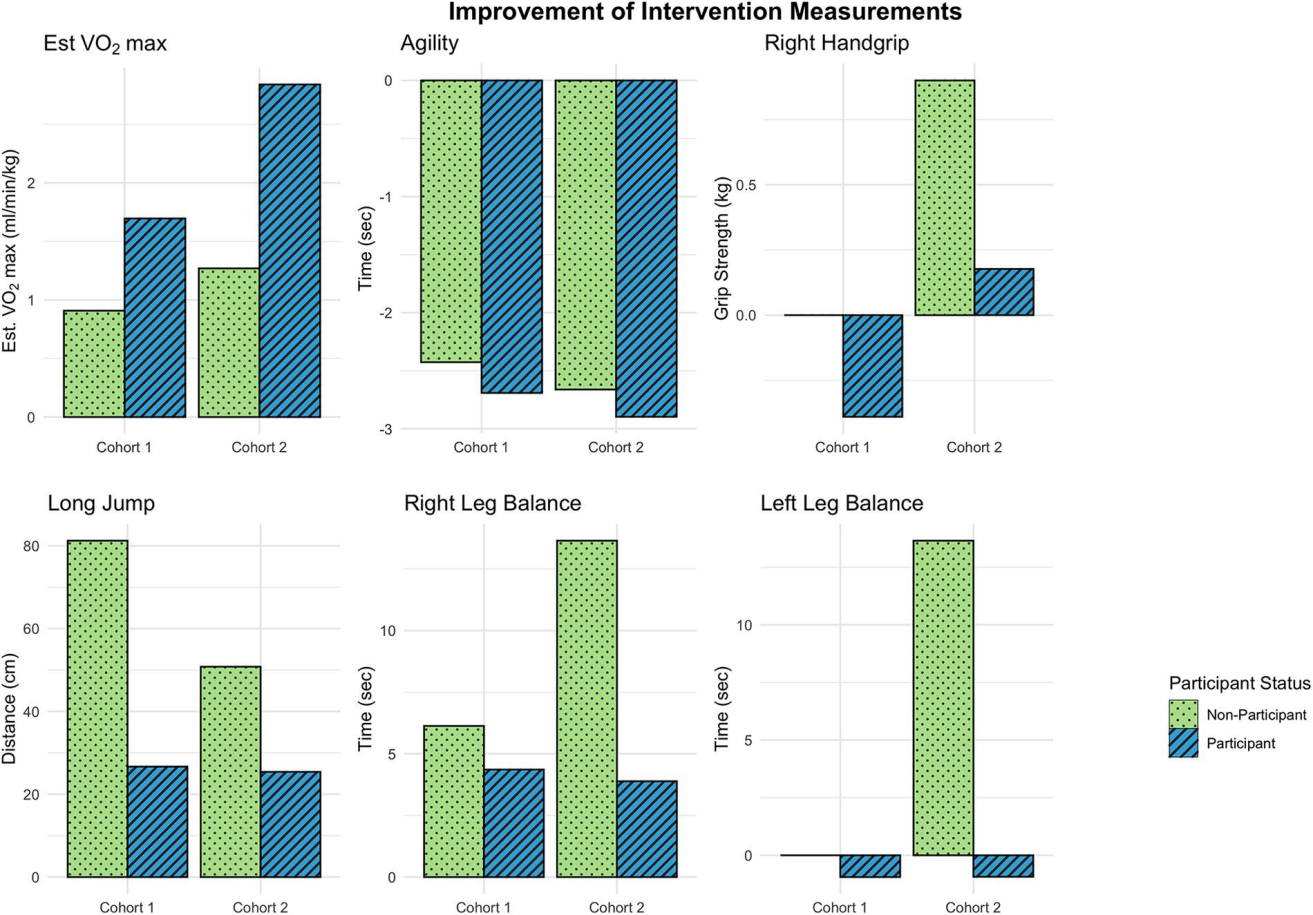
Differences between baseline and end of study physical activity measurements separated by participation and cohort. Each measurement (Estimated VO_2_, Agility, Right Handgrip, Long Jump, Right and Left Leg Balance) presents Cohort 1 on the left with non-participants in green dots and participants in blue stripes.

In the low participation groups, C2 demonstrated greater gains in VO₂, agility, right grip strength, and leg balance. C1 showed more improvement in left grip strength. However, none of these differences were statistically significant (*p* > 0.05) (Table 4, Figure 2).

**Table 4:** Physical activity measurements differences between baseline and end of study by participation and cohort.

Characteristic	Participants					Non-participants				
	Cohort 1 *N* = 5^a^ (95% CI)	Cohort 2 *N* = 10^a^ (95% CI)	Difference (95% CI)^a^	*p*-value	Effect size^b^	Cohort 1 *N* = 5^a^	Cohort 2 *N* = 10^a^	Difference (95% CI)^a^	*p*-value	Effect Size^b^
Estimated VO_2_ (mL/min/kg)	1.70 (−0.30, 3.70)	2.84 (−0.27, 5.95)	−1.14 (−2.22, 4.51)	0.8	0.12	0.91 (0.50, 1.32)	1.27 (−0.31, 2.85)	−0.36 (−1.24, 1.96)	0.3	0.30
Agility (s)	−2.69 (−5.23, −0.16)	−2.95 (−3.77, −2.13)	0.26 (−2.74, 2.22)	0.5	0.24	−2.43 (−3.30, −1.55)	−2.66 (−4.08, −1.24)	0.24 (−1.79, 1.32)	>0.9	0.00
Right Handgrip (kg)	−0.39 (−2.62, 1.84)	0.05 (−0.86, 0.96)	−0.44 (−1.75, 2.63)	0.8	0.12	−0.00 (−2.16, 2.16)	0.90 (−0.22, 2.02)	−0.90 (−1.36, 3.16)	0.3	0.34
Left Handgrip (kg)	−1.16 (−2.00, −0.32)	0.62 (0.00, 1.24)	−1.78 (0.87, 2.69)	0.003	0.92	1.16 (−0.78, 3.11)	0.96 (−0.98, 2.90)	0.20 (−2.70, 2.29)	0.9	0.06
Long Jump (cm)	−7.12 (−53.03, 38.79)	−8.38 (−21.20, 4.44)	1.26 (−46.29, 43.77)	0.6	0.18	30.66 (−2.62, 63.94)	3.75 (−18.18, 25.67)	26.91 (−63.43, 9.60)	0.2	0.43
Right Leg Balance (sec)	4.36 (−15.48, 24.20)	4.28 (−2.96, 11.51)	0.08 (−19.52, 19.35)	>0.9	0.00	6.13 (−7.95, 20.21)	13.65 (0.80, 26.49)	−7.52 (−9.79, 24.82)	0.3	0.34
Left Leg Balance (sec)	−0.95 (−29.14, 27.25)	−1.03 (−13.92, 11.87)	0.08 (−27.94, 27.78)	0.8	0.12	0.00 (NaN, NaN)	13.66 (−2.09, 29.40)	−13.66 (−2.09, 29.40)	0.030	0.60

CI, confidence interval.

a Difference in mean change scores between cohorts (95% CI).

b Wilcoxon effect size (unpaired).

### 3.3 Timepoint-based comparisons

At baseline, C2 outperformed C1 in estimated VO₂ [Δ = 6.53; (4.23,8.82); Wilcoxon Effect Size 0.87; *p* < 0.001] and right-hand grip strength [Δ = 2.96; (0.42,5.49); Wilcoxon Effect Size 0.45; *p* = 0.023], while C1 performed better in one-leg balance [right: Δ = −18.06; [−31.09,−5.03]; Wilcoxon Effect Size 0.58; *p* = 0.006; left: Δ = −24.36; [−35.16,−13.56]; Wilcoxon Effect Size 0.71; *p* ≤ 0.001]. When stratified by participation, both C2 participants and non-participants had significantly higher estimated VO₂ scores [Δ = 5.49; (2.23,8.74); Wilcoxon Effect Size 0.68; *p* = 0.036 and Δ = 7.58; (3.95,11.22); Wilcoxon Effect Size 1.00; *p* < 0.001, respectively]. C1 non-participants performed better in one-leg balance [right: Δ = −22.97; (−39.84,−6.10); Wilcoxon Effect Size 0.73; *p* = 0.013; left: Δ = −26.03; (−40.1,−12.0); Wilcoxon Effect Size 0.80; *p* = 0.004]. All time-based comparisons can be found in Supplementary Tables S1, S2.

At study completion, C2 continued to outperform C1 in VO₂ [Δ = 7.34; (4.52,10.16); Wilcoxon Effect Size 0.90; *p* < 0.001] and right-hand grip [Δ=3.59; (1.14,6.04); Wilcoxon Effect Size 0.56; *p* = 0.009]. C1 showed significantly better results in one-leg balance [right: Δ = −14.49; (−27.13,−1.85); Wilcoxon Effect Size 0.46; *p* = 0.025; left: Δ = −17.65; (−28.08,−7.22); Wilcoxon Effect Size 0.60; *p* = 0.003]. Stratified results showed C2 participants and non-participants had significantly better VO₂ scores [Δ=6.63; (2.72,10.54); Wilcoxon Effect Size 0.80; *p* = 0.016 and Δ = 7.94; (3.04,12.85); Wilcoxon Effect Size 1.0; *p* < 0.001, respectively]. C2 non-participants also had stronger right-hand grip [Δ = 5.54; (2.23,8.85); Wilcoxon Effect Size 0.89; *p* = 0.001], while C1 non-participants excelled in long jump [Δ = −34.35; (−63.28,−5.41); Wilcoxon Effect Size 0.69; *p* = 0.019]. C2 participants outperformed C1 participants in left leg balance [Δ = −12.38; (−25.33,0.58); Wilcoxon Effect Size 0.50; *p* = 0.040].

### 3.4 Health knowledge

Health knowledge scores showed no significant improvement from baseline to study end. The average score rose modestly from 63% to 66% [95% CI (−0.65, 8.1); Wilcoxon Effect Size 0.11; *p* = 0.62]. However, differences in cohort and control question responses influenced this comparison. Control question scores were significantly different (C2: 52%; C1: 79%), suggesting a disparity in reading levels. Baseline scores were higher in C1 (71%) than C2 (57%) (Wilcoxon Effect Size 0.64; *p* < 0.001), and this trend persisted at study end (C1: 72%; C2: 62%; Wilcoxon Effect Size 0.5; *p* = 0.02).

**Figure 3:**
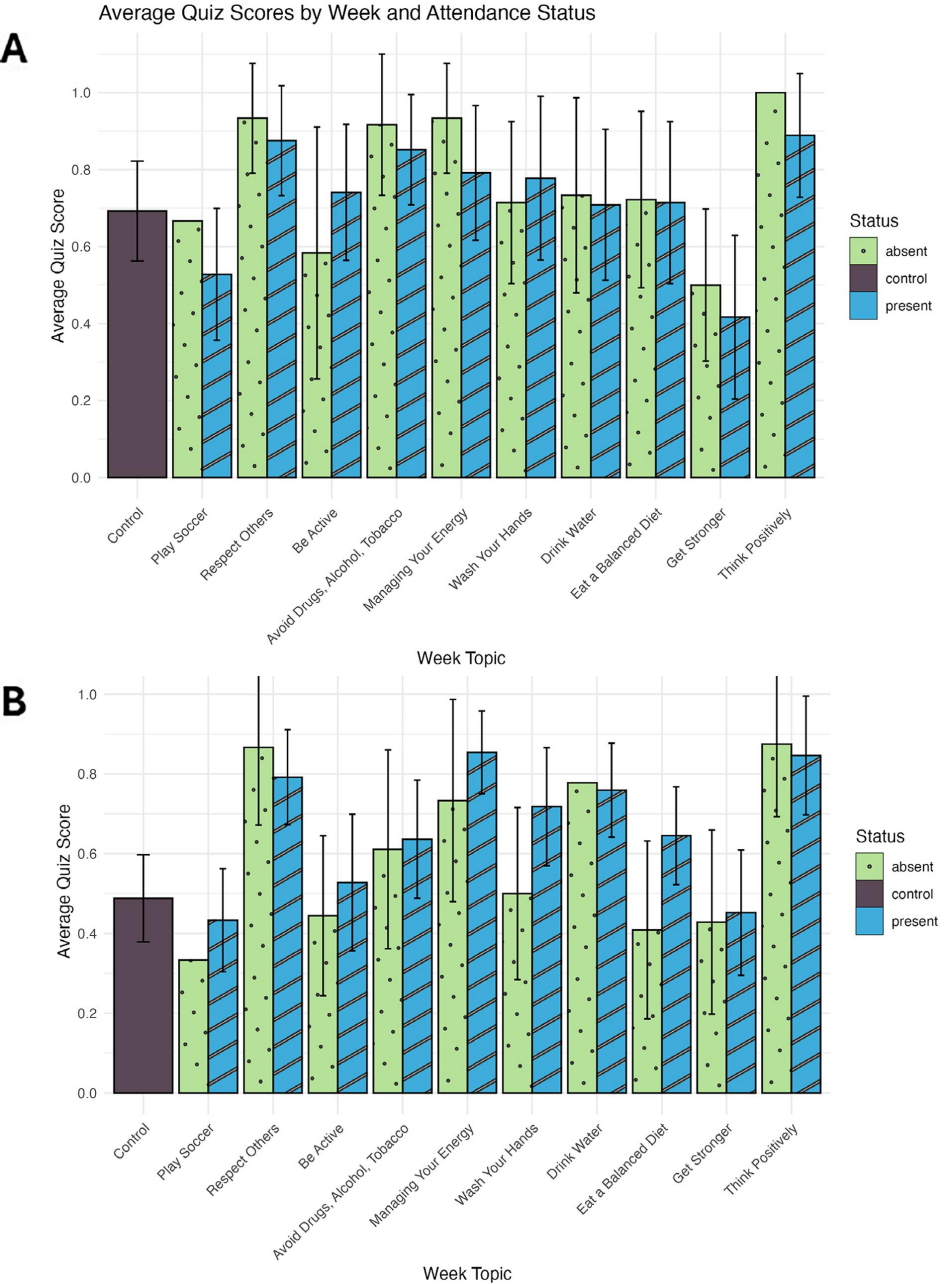
Average health knowledge score by topic based on attendance and participation when the weekly topic was discussed. The *y* axis states the Health Knowledge score from zero to 100% on a scale. The *x* axis includes the average of the questions associated with the weekly topics and controls. The control questions are a set of control questions that students should be able to answer without having the program. The legend on the right labels those present as blue stripes and those absent as green dots. Solid brown represents control questions. Panel **A** is Cohort 1 and Panel **B** is Cohort 2.

Figure 3 displays average end-of-study quiz scores by topic. No significant differences were found between participants and non-participants, though C2 showed greater variation between select topics (e.g., “Wash your hands” and “Eating a balanced diet”).

It's worth noting that health knowledge topics were covered during C1's second weekly session (Wednesdays), often at the end. This allowed some children to be present for the review even if they didn't actively participate (e.g., band/chorus practice).

## 4 Discussion

This pilot study represents the first implementation of the “11 for Health” program in the United States, aimed at evaluating its feasibility for upper elementary school children participating in after-school programs. Findings suggest that while the 11-week program was feasible to conduct based on session delivery and study measurement completion, its suitability for this specific chosen population may be limited due to variability in after-school program support. Analysis of pre- and post-intervention surveys and fitness assessments yielded mixed results. While some improvements in physical fitness were noted over time, there were no significant gains in the students' health knowledge. C2 did perform better on some physical fitness measurements, but C1 performed better on the health knowledge test, but this could be due to differences in reading ability overall and language barriers.

Physical fitness was a secondary preliminary outcome in this study, involving assessments of agility, estimated VO_2_, handgrip strength, long jump distance, and standing balance. Previous studies, including “FIFA 11 for Health” and its adaptations, have consistently reported positive outcomes in physical fitness assessments (11, 12, 24–28). Specifically, there was a statistically significant improvement in agility in both participation groups in both cohorts. Most studies employed a 20- to 30-meter sprint to evaluate agility, with all demonstrating significant improvements in speed among participants (28, 29). In contrast, grip strength did not show significant changes post-intervention in C1 but did in C2, with high participants showing more improvement. This finding aligns with existing literature, which indicates that upper limb muscular strength can vary greatly depending on the sport played and the age of the participant (30, 31). Given that the study lasted only 10–11 weeks, it is plausible that this duration was insufficient for participants to develop the upper body strength necessary for significant improvements in handgrip strength. Other research focusing on different forms of physical exercise over longer periods has shown significant changes in strength levels (31, 32).

The long jump distance increased slightly by an average of 4 cm overall from baseline; however, this change was not statistically significant. While these findings were insignificant, they are consistent with results from similar studies (25, 28). For example, a Danish adaptation of the program reported no change in long jump distance from baseline assessments, mirroring our outcomes (29). Although our study did not show significant changes in the long jump, some studies show improvements in this assessment after a similar 12-week program (33). Postural balance test results increased slightly from pre- to post-intervention across both groups. Although children's ability to balance on one leg did not change significantly, they balanced for a longer duration compared to participants in the “11 for Health” program conducted in China (28). The postural balance is also significant in showing improvement in muscular skeletal fitness; adaptations to the 11 for Health program have shown improvements (11, 25, 26, 31).

C1 scored higher on the knowledge test but showed no improvement at end of study. C2 showed a trend of improvement, but it did not achieve significance. Differences in reading abilities between the two schools may have influenced comprehension of program materials and confounded knowledge outcomes. C2's after-school program servesmany Hispanic participants who spoke English as a second language. Analysis of knowledge outcomes revealed variability based on attendance throughout the 11 weeks. Higher average scores were observed for absent students in weeks 1, 2, 4, 5, 10, and 11 (only week 2 for C2), while weeks 3 and 6 favored students present in C1 and weeks 1, 3, 5, 6, and 8 in C2. Some of these topics that favored absence include information participants may have learned in other programs or in school (e.g., Respect Others, Avoid Drugs and Alcohol, Think Positively). Overall changes in pre- and post-questionnaire scores were not statistically significant,. Despite these results, previous Danish studies have demonstrated that adaptations of health education can lead to significant improvements in health knowledge scores (13, 29).

When evaluating feasibility, one major challenge encountered was inconsistent attendance. As the program was conducted in an after-school setting, children often arrived hyperactive, fatigued, and/or easily distracted. This environment allowed frequent early pickups and competing activities, reducing the likelihood of consistent participation. To address these challenges, several strategies could be considered. Enhancing parental engagement may contribute to fostering a positive attitude towards attendance, as noted in the literature on after-school interventions (34). Integrating the program into the school curriculum, as successfully demonstrated in other implementations of “11 for Health”, may also improve participation rates (10, 17, 18, 20–23, 27–30).

Another challenge seen in C1 only was the type of after-school program. This after-school program offers a choice-based curriculum. This means that the participants attending could choose their activities for the day (outside of homework time). This structure posed challenges when recruiting participants and communicating the research study objectives, as introducing the program required temporarily limiting some of their usual activity choices. This environment fostered feelings of jealousy among participants who were required to engage in the study rather than freely choose their pursuits. Such feelings often led to complaints and withdrawal from participation, with some parents removing their children from the study altogether. Additionally, scheduling conflicts with the after-school chorus and band resulted in three students arriving late on certain days. While this did not affect the educational components, it detracted from the physical aspects of the program. Consequently, this restriction may have hindered participant engagement as we were often competing with other games and playground free play.

Furthermore, the pilot was directed by researchers rather than elementary educators. While instructors were familiar with the program protocol and trained in the activities, they lacked specific training in effectively engaging elementary-aged students. The instructors in C2 were more familiar with the participants because they had previously implemented a reading program with the participants, but still not formally trained as elementary educators. This challenge highlighted the need for future implementations to include trained coaches with experience in child engagement techniques, similar to those utilized in other adapted programs (12, 24, 27, 28, 35–37). Many of these conducted the 11 for Health program during school time by the participants' teachers. Additional limitations included the small sample size and variable participation rate and the absence of a control group. Unlike numerous studies that have integrated “11 for Health” into compulsory physical education classes, our program was delivered during an after-school period where children had the autonomy to choose their activities or needed to prioritize academics. Consequently, this structure led to reduced participation, as some children opted not to engage with the program. This could have also played a role in the measurement variability.

Despite these challenges, this pilot study of the “11 for Health” program has several notable strengths. A significant strength was the successful adaptation of a weight-neutral approach to the intervention. Since the original program's launch in Africa in 2009 and its subsequent adaptations in various regions, including Latin America (33, 38), the Caribbean, Southeast Asia (28), Oceania, and Denmark (24, 25, 27, 37), the protocol has been modified to accommodate diverse populations and languages. Unlike other programs targeting obesity and weight loss (39), our initiative focused on minimizing weight stigma by reframing terms from “Controlling Your Weight” to “Managing Your Energy”, for example. This approach emphasized promoting healthy behaviors and body positivity without making children self-conscious about their bodies. Utilizing a weight-neutral framework posits numerous benefits, such as fostering a healthy relationship between food and exercise and potentially mitigating the risk of developing eating disorders or compulsive exercise behaviors in the future (40).

Additionally, a strength of our adaptation was the recognition of cultural differences and contextual sensitivity in curriculum implementation among American children. For instance, one lesson required students to hold hands during activities, an expectation that did not align with the comfort levels of the participants. This led to visible reluctance, prompting a necessary pivot to ensure that the intended lesson could still be effectively taught without compromising student comfort. During the second cohort, a penny was used instead of hands, where each participant held on to each end and this was better accepted.

Despite challenges regarding attendance and participation, the study successfully completed all measurements for each enrolled student. It is noteworthy that similar programs in other countries leveraged physical education classes to deliver the intervention. However, our program's implementation in an after-school context proved more challenging due to the necessity of providing children with activity choices. C2 was not a choice-based intervention, but academic tutoring took precedence over physical activity; therefore, attendance was still a challenge for some participants.

Moreover, this pilot provided evidence that the program is logistically feasible in the United States, albeit with necessary modifications. The findings indicate that it is possible to offer structured health and physical activity interventions in after-school programs that maintain a choice-based curriculum with the right preparation. C2 provided on-site counselors that attended every session, unlike C1. The additional help with participant attention was appreciated and significantly increased the participants cooperation with instructions.

In conclusion, participants in both cohorts were able to improve their cardiorespiratory fitness and agility, demonstrating the potential benefits of the program regardless of background or prior experience. These findings should be interpreted cautiously in light of the 69% attendance rate and the absence of significant gains in health knowledge. This American pilot of “*11 for Health*” provides evidence that implementing the program in an after-school setting is feasible when using a culturally adapted and appropriately translated Danish protocol. While feasibility was confirmed, the intervention may yield more pronounced outcomes in populations with limited access to extracurricular sports. Future implementations could benefit from integration into school curricula or partnerships with community organizations that have the necessary resources and personnel. Further research is needed to assess both the short- and long-term effects of the program.

## Data Availability

The original contributions presented in the study are included in the article/Supplementary Material, further inquiries can be directed to the corresponding author.
