# Supplementary material for: Beyond the game: evaluating a soccer-centered approach to physical activity and health education in children
Source: Front Sports Act Living. 2026 Jul 15;8:1765167. doi: 10.3389/fspor.2026.1765167 (PMC13417902; doi:10.3389/fspor.2026.1765167)
Supplement: Supplementary file 1 [file Supplementaryfile1.docx]

Supplementary Material

Supplement Table 1. Baseline Physical Activity Measurements by Participants and Cohort

| Characteristic | Participants | | | | | Non-Participants | | | | |
| --- | --- | --- | --- | --- | --- | --- | --- | --- | --- | --- |
|  | Cohort 1 N=5^1^ (95% CI) | Cohort 2 N=10^1^ (95% CI) | Difference (95% CI)^2^ | p-value^3^ | Effect Size^4^ | Cohort 1 N=5^1^ (95% CI) | Cohort 2 N=10^1^ (95% CI) | Difference (95% CI)^2^ | p-value^3^ | Effect Size^4^ |
| Estimated VO_2_ (ml/min/kg) | 17.52 (0.87) | 23.01 (4.47) | 5.49 (2.23, 8.74) | 0.036 | 0.68 | 17.64 (1.36) | 25.22 (4.95) | 7.58 (3.95, 11.22) | <0.001 | 1.00 |
| Agility (sec) | 11.27 (1.42) | 12.03 (1.42) | 0.76 (-1.03,2.55) | 0.440 | 0.28 | 10.64 (0.84) | 11.29 (2.07) | 0.65 (-0.92, 2.22) | 0.475 | 0.23 |
| Right Handgrip (kg) | 12.24 (3.24) | 13.60 (3.18) | 1.36 (-2.72, 5.43) | 0.624 | 0.18 | 12.77 (2.44) | 17.42 (4.68) | 4.64 (0.91, 8.38) | 0.010 | 0.74 |
| Left Handgrip (kg) | 13.29 (5.14) | 12.33 (3.16) | -0.97( -7.21, 5.28) | 0.759 | 0.12 | 12.29 (2.78) | 15.22 (3.94) | 2.93 (-0.55, 6.40) | 0.133 | 0.46 |
| Long Jump (cm) | 170.18 (28.30) | 145.29 (16.28) | -24.89 (-59.30, 9.52) | 0.096 | 0.56 | 160.02 (33.50) | 152.59 (28.99) | -7.43 (-41.56, 26.70) | 0.669 | 0.14 |
| Right Leg Balance (sec) | 39.60 (18.81) | 28.64 (18.65) | -10.96 (-34.64, 12.71) | 0.296 | 0.36 | 52.71 (14.24) | 29.75 (18.30) | -22.97 (-39.84, -6.10) | 0.013 | 0.73 |
| Left Leg Balance (sec) | 50.00 (14.14) | 28.98 (20.77) | -21.02 (-41.00, -1.04) | 0.084 | 0.58 | 60.00 (0.00) | 33.97 (19.63) | -26.03 (-40.08, -11.99) | 0.004 | 0.80 |

| *^1^*Mean (SD) |
| --- |
| *^2^*Difference in means (95% Confidence Interval) |
|  |
| *^3^*Wilcoxon rank sum test; Wilcoxon rank sum exact test |
| *^4^*Wilcoxon Effect Size (Unpaired) |

Abbreviation: CI=Confidence Interval

Supplement Table 2. End of Study Physical Activity Measurements by Participants and Cohort

| Characteristic | Participants | | | | | Non-participants | | | | |
| --- | --- | --- | --- | --- | --- | --- | --- | --- | --- | --- |
|  | Cohort 1 N=5^1^ (95% CI) | Cohort 2 N=10^1^ (95% CI) | Difference (95% CI)^2^ | p-value^3^ | Effect Size^4^ | Cohort 1 N=5^1^ (95% CI) | Cohort 2 N=10^1^ (95% CI) | Difference (95% CI)^2^ | p-value^3^ | Effect Size^4^ |
| Estimated VO_2_ (ml/min/kg) | 19.22 (2.42) | 25.85 (4.57) | 6.63( 2.72, 10.54) | 0.016 | 0.80 | 18.55 (1.60) | 26.49 (6.73) | 7.94 (3.04, 12.85) | <0.001 | 1.00 |
| Agility (sec) | 8.57 (0.70) | 9.08 (0.99) | 0.50 (-0.47, 1.47) | 0.440 | 0.28 | 8.21 (0.69) | 8.63 (1.60) | 0.41 (-0.81, 1.64) | 0.601 | 0.17 |
| Right Handgrip (kg) | 11.85 (2.44) | 13.65 (3.76) | 1.80 (-1.73, 5.32) | 0.371 | 0.32 | 12.77 (2.06) | 18.32 (4.21) | 5.54 (2.23, 8.85) | 0.001 | 0.89 |
| Left Handgrip (kg) | 12.13 (4.82) | 12.95 (2.85) | 0.82 (-5.05, 6.68) | 0.679 | 0.16 | 13.46 (2.70) | 16.18 (3.93) | 2.72 (-0.70, 6.15) | 0.161 | 0.43 |
| Long Jump (cm) | 163.06 (45.03) | 136.91 (23.87) | -26.15 (-80.93, 28.62) | 0.244 | 0.40 | 190.68 (29.55) | 156.34 (21.17) | -34.35 (-63.28, -5.41) | 0.019 | 0.69 |
| Right Leg Balance (sec) | 43.96 (23.31) | 32.91 (16.17) | -11.05 (-39.46, 17.37) | 0.323 | 0.34 | 58.84 (3.06) | 43.39 (18.69) | -15.45 (-28.93, -1.97) | 0.098 | 0.43 |
| Left Leg Balance (sec) | 49.06 (15.74) | 27.95 (11.39) | -21.10 (-40.31, -1.89) | 0.031 | 0.72 | 60.00 (0.00) | 47.62 (18.11) | -12.38 (-25.33, 0.58) | 0.040 | 0.50 |

| *^1^*Mean (SD) |
| --- |
| *^2^*Difference in means (95% Confidence Interval) |
|  |
| *^3^*Wilcoxon rank sum test; Wilcoxon rank sum exact test |
| *^4^*Wilcoxon Effect Size (Unpaired) |

Abbreviation: CI=Confidence Interval


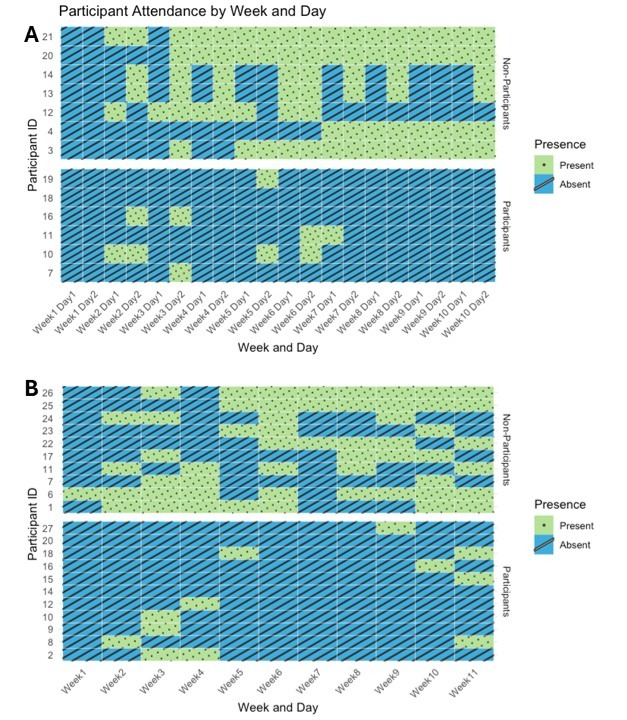


**Supplementary Figure 1.** Intervention Attendance by Cohort. Panel A is Cohort 1, where the intervention was delivered twice a week for 45 minutes and Panel B is Cohort 2, where the intervention was delivered once a week for 90 minutes. Absent is represented by green dots and Present is represented by blue stripes. The y axis is each study ID and the x axis is the session.
